# Ivy leaves extract EA 575 in the treatment of cough during acute respiratory tract infections: meta-analysis of double-blind, randomized, placebo-controlled trials

**DOI:** 10.1038/s41598-022-24393-1

**Published:** 2022-11-21

**Authors:** Andreas Völp, Jennifer Schmitz, Michael Bulitta, Esther Raskopf, Cengizhan Acikel, Ralph Mösges

**Affiliations:** 1 Psy Consult Scientific Services, Hamburg, Germany; 2 CRM Biometrics GmbH, Rheinbach, Germany; 3 ClinCompetence Cologne GmbH, Theodor-Heuss-Ring 14, 50668 Cologne, Germany; 4 Institute of Medical Statistics and Computational Biology (IMSB), Medical Faculty, University at Cologne, Cologne, Germany

**Keywords:** Medical research, Respiratory tract diseases

## Abstract

Ivy leaves extracts have been used successfully to treat acute cough, and data from well-controlled trials is accumulating. We present a meta-analysis of two double-blind, randomized, placebo-controlled trials. Patients with acute respiratory tract infection (ARTI) received ivy leaves dry extract EA 575 (n = 228) or placebo (n = 162) for 7 days, followed by a 7-day period without treatment. The main efficacy outcome was the Bronchitis Severity Score (BSS). Individual patient data meta-analyses were performed using mixed models for repeated measures, analysis of covariance and logistic ordinal regression. Significant BSS differences between EA 575 and placebo occurred already after 2 days and increased until treatment end, with BSS reductions of 8.6 ± 0.2 and 6.2 ± 0.2 (marginal means ± SEM; p < 0.001). The score reduction for placebo after 7 days was comparable to that for EA 575 after 4 days. In the EA 575 group, the proportion of cough-free patients was 18.1% at treatment end and 56.2% at end of follow-up, compared to 9.3% and 25.6% for placebo, respectively. Adverse event rates for EA 575 and placebo were comparable. EA 575 reduces effectively the intensity of acute cough associated with ARTIs and leads to a significant acceleration of recovery. No safety signals were observed.

## Introduction

Acute respiratory tract infections (ARTIs) are the most prevalent diseases in primary care^1–3^. An estimated 90% of such infections have a viral etiology^4–6^, and thus antibiotic treatment, even though quite commonly prescribed, is usually not indicated^7–9^. On average, adults suffer from 2–3 ARTIs per year^10^. While most ARTIs are trivial and self-limiting^11^, they may nevertheless cause significant suffering and impairment of every-day activities^12^, including reduced productivity or absence from work. Their economic impact through direct and indirect costs is therefore enormous^13,14^.

Cough is one of the principal symptoms of ARTIs such as acute bronchitis or the common cold. Along with mucociliary clearance, it is an important defensive mechanism of the organism in response to stimuli irritating nerve terminals located in the mucous membrane of the upper airways^15,16^. In healthy individuals, mucus traps inhaled particles while mucociliary clearance continuously drains out the mucus produced, thereby minimizing contact of external agents with the pulmonary epithelium. In ARTIs, cough may either be unproductive (dry, no excretion) or productive (wet, expectoration of mucus). Productive cough should not be suppressed as it enables the evacuation of mucus from the bronchi^16^.

The features and duration of cough depend on its etiology and are influenced by environmental factors^17^. Whereas most symptoms of ARTIs subside completely within two weeks even untreated, cough in particular may persist for more than three weeks^18^. As no curative antiviral treatment exists yet, symptomatic treatment is motivated by a reduction of the disease burden and by the prevention of more serious complications such as bacterial superinfections or pneumonia^19^.

Today’s over-the-counter (OTC) market offers a wide range of antitussive agents and expectorants for symptomatic cough treatment even though the efficacy of many of these treatments is still a matter of debate, not least due to methodological issues of available studies^20–22^. Extracts from dried ivy leaves (*Hedera helix* L.) are among the most widely used expectorants that act by improving mucus excretion. EA 575 (trade name: Prospan) is a liquid formulation containing dry extract from *Hedera helix* L. It is marketed as a medicinal product in different countries for improving complaints of chronic-inflammatory bronchial diseases and for treatment of acute inflammations of the respiratory tract accompanied by coughing. The multi-component mixture EA 575 contains e.g., saponins, flavonoids, and phenolic acids^23–25^. The extract exerts an anti-inflammatory effect that has been linked to an inhibition of the transcriptional activity of NF-κB (nuclear factor κ-light-chain-enhancer of activated B-cells) and a subsequent decrease in the cellular release of the cytokine IL-6^26,27^.

A large number of interventional and non-interventional studies investigating the effects of ivy leaves extracts have been published. For EA 575, data from more than 65,000 patients are available from clinical trials and post-marketing research^22,28^. The results indicate that in patients with ARTIs, EA 575 reduces the frequency and duration of coughing, and thus also ameliorates the disease burden experienced by the patients subjectively. The updated systematic review of Sierocinski et al.^22^ also includes evidence from two double-blind, randomized, placebo-controlled trials assessing the efficacy EA 575 in ARTIs^29,30^, which the authors identified as the only investigations of this type of an ivy leaves dry extract mono-preparation that complied with currently applicable scientific standards. However, the authors neither performed a meta-analysis of these trials, nor did they identify any other work that provided a quantitative synthesis of studies performed for ivy leaf extract preparations.

We present the results of an individual patient data (IPD) meta-analysis of the two randomized, double-blind, placebo-controlled trials^29,30^, both of which confirmed the efficacy of EA 575 individually. The studies were performed in similar populations of adult patients suffering from ARTI characterized by coughing and according to essentially similar protocols.

## Results

### Disposition of patients and baseline characteristics

**Figure 1:**
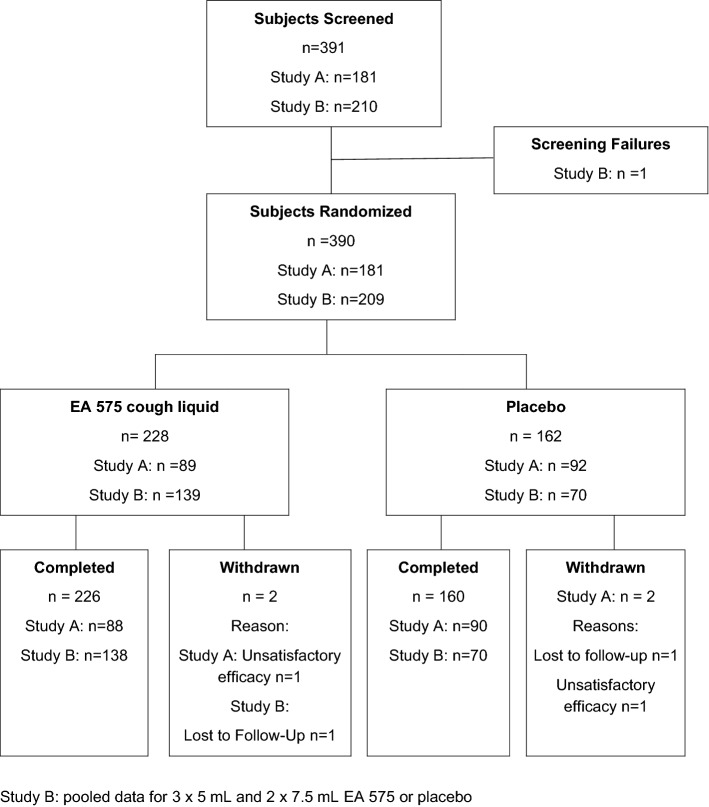
Disposition of patients.

Studies A and B were performed between 2015 and 2017 in general and ENT practices in Germany (five sites per study). A total of 391 patients were screened, 390 were randomized, and 386 completed the studies as scheduled (Fig. 1). Reasons for withdrawal were unsatisfactory efficacy (one patient in each treatment group) and loss to follow-up (one in each group). All randomized patients were analyzed for efficacy and safety.

The study participants’ demographic and baseline characteristics are shown in Table 1. The studies, as well as the treatment groups within each study, were essentially comparable with regard to age, sex, and basic anthropometric measures. Baseline comparability was also assured for all cough-related outcomes (Table 1).

**Table 1:** Patient baseline characteristics (full analysis set; means ± SD or absolute frequency and %).

	Study A		Study B		Pooled data	
	EA 575 (n = 89)	Placebo (n = 92)	EA 575 (n = 139)	Placebo (n = 70)	EA 575 (n = 228)	Placebo (n = 162)
Age (years)	36.2 ± 14.6	36.4 ± 13.4	36.4 ± 12.8	34.9 ± 12.7	36.3 ± 13.5	35.8 ± 13.1
**Sex**						
Male	49 (55.1%)	44 (47.8%)	67 (48.2%)	36 (51.4%)	116 (50.9%)	80 (49.4%)
Female	40 (44.9%)	48 (52.2%)	72 (51.8%)	34 (48.6%)	112 (49.1%)	82 (50.6%)
Height (cm)	175.5 ± 9.3	173.9 ± 10.5	174.2 ± 10.2	174.3 ± 8.6	174.7 ± 9.8	174.0 ± 9.7
Weight (kg)	79.5 ± 16.9	78.5 ± 17.3	78.0 ± 17.3	79.2 ± 16.3	78.6 ± 17.1	78.8 ± 16.8
BSS total score (points)^a^	11.2 ± 1.5	11.3 ± 1.3	11.8 ± 1.6	11.8 ± 1.5	11.6 ± 1.6	11.5 ± 1.4
Cough severity VAS (mm)^a^	72.8 ± 8.9	72.3 ± 10.0	73.4 ± 8.0	72.1 ± 7.6	73.2 ± 8.3	72.2 ± 9.0
Cough frequency VRS (points)^a^	3.3 ± 0.6	3.4 ± 0.7	3.5 ± 0.6	3.6 ± 0.6	3.4 ± 0.6	3.5 ± 0.6

*BSS* Bronchitis Severity Score, *VAS* Visual analog scale, *VRS* Verbal rating scale.

^a^Higher scores indicate more pronounced impairment.

### Bronchitis Severity Score

**Figure 2:**
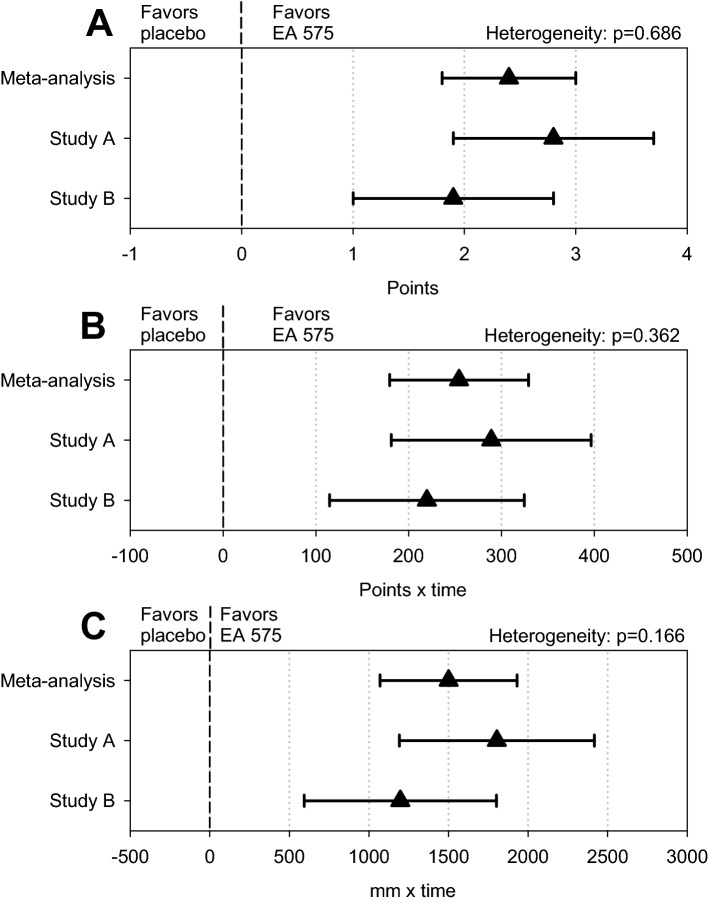
Forest plots of meta-analysis main results (full analysis set; marginal means and 95% confidence intervals as well as p-value for heterogeneity). (**A**) Bronchitis Symptom Score, Day 7 (treatment end); (**B**) Bronchitis Symptom Score, AUC_Baseline_—Day 7; (**C**) Cough Severity VAS, AUC_Baseline_—Day 7.

In both treatment groups, the BSS total score showed a monotonic decrease between baseline and the end of follow-up, which was significantly more pronounced in the EA 575 group (Table 2). For BSS total score decrease, a statistically significant treatment group difference by 0.9 points was observed already after 2 days of treatment while a difference by 2.4 points was observed at treatment end after one week, with a minimum advantage of 1.8 points favoring EA 575 according to the associated 95% confidence interval (CI; Fig. 2A). Consequently, a treatment effect of comparable magnitude was also observed for the AUC determined for the entire 7-day treatment period (Fig. 2B). Moreover, both figures show that a significant effect of EA 575 beyond that of placebo was not only observed in the meta-analysis but also in Studies A and B individually. The treatment group difference tended to decrease slightly during the follow-up week after the end of treatment (even though it was still statistically significant at end of follow-up), which may have been attributable to the natural course of the disease and increasing spontaneous remission in the placebo group.

**Table 2:** Bronchitis Severity Score—meta-analysis and individual trial results (full analysis set; marginal means ± SEM for EA 575 and placebo, difference between marginal means and 95% confidence interval, p-value derived from treatment-by-visit interaction).

Treatment day/period	EA 575 (n = 228)	Placebo (n = 162)	Difference (95% CI)	p
**Meta-analysis**				
2	2.3 ± 0.2	1.4 ± 0.2	0.9 (0.5; 1.4)	< 0.001
3	4.5 ± 0.2	2.7 ± 0.2	1.8 (1.2; 2.3)	< 0.001
4	6.1 ± 0.2	4.1 ± 0.2	2.0 (1.4; 2.7)	< 0.001
7 (treatment end)	8.6 ± 0.2	6.2 ± 0.2	2.4 (1.8; 3.0)	< 0.001
14 (follow-up)	10.5 ± 0.1	8.9 ± 0.2	1.5 (1.1; 2.0)	< 0.001
AUC baseline—Day 7	1157.4 ± 24.8	1411.7 ± 28.9	254.3 (179.5; 329.2)	< 0.001

Treatment day/period	EA 575 (n = 89)	Placebo (n = 92)	Difference (95% CI)	p
**Trial A**				
2	2.2 ± 0.2	1.1 ± 0.2	1.1 (0.5; 1.7)	< 0.001
3	4.3 ± 0.3	2.4 ± 0.3	1.9 (1.1; 2.8)	< 0.001
4	5.9 ± 0.3	3.6 ± 0.3	2.2 (1.3; 3.2)	< 0.001
7 (treatment end)	8.5 ± 0.3	5.7 ± 0.3	2.8 (1.9; 3.7)	< 0.001
14 (follow-up)	10.3 ± 0.2	8.8 ± 0.2	1.5 (0.9; 2.2)	< 0.001
AUC Baseline—Day 7	1160.2 ± 38.9	1449.2 ± 38.1	289.0 (182.4–395.6)	< 0.001

Treatment day/period	EA 575(n = 139)	Placebo (n = 70)	Difference (95% CI)	p
**Trial B**				
2	2.4 ± 0.2	1.7 ± 0.3	0.7 (0.0; 1.3)	0.047
3	4.5 ± 0.2	3.0 ± 0.3	1.5 (0.2; 2.3)	< 0.001
4	6.3 ± 0.3	4.7 ± 0.4	1.6 (0.8; 2.5)	< 0.001
7 (treatment end)	8.6 ± 0.2	6.7 ± 0.3	1.9 (1.0; 2.7)	< 0.001
14 (follow-up)	10.6 ± 0.2	9.1 ± 0.3	1.5 (0.9; 2.1)	< 0.001
AUC baseline—Day 7	1154.6 ± 31.0	1374.2 ± 43.7	219.6 (114.6–324.6)	< 0.001

*CI* Confidence interval, *SEM* standard error of the mean.

BSS, observed values: score points; AUC: score points × hours.

For the meta-analysis treatment group comparison of intra-individual change of the BSS total score between baseline and treatment end, Hedges’ g was determined as g = 0.77, corresponding to a moderate to large treatment effect according to the conventional interpretation^31^.

Based on the criteria proposed by the German Institute for Quality and Efficiency in Health Care, a detectable decrease of at least 4 points between the BSS total scores at baseline and at treatment end was achieved by 212/227 patients (93.4%) treated with EA 575 and by 121/160 patients (76.5%) in the pooled data set of both trials (p < 0.001). Proportions in trials A and B were similar to those in the pooled data, and the treatment group difference was also significant for each trial analyzed separately (p < 0.01).

### Cough severity VAS

For the cough severity VAS, the AUC determined for the 7-day treatment phase was determined in the meta-analysis as 8176 ± 142 score points/hour for EA 575 and as 9677 ± 166 score points/hour for placebo, resulting in a treatment group difference of 1501 (95% CI 1070; 1932) score points/hour (p < 0.001; Fig. 2C; lower scores indicate less pronounces cough severity). On an individual study level, treatment group AUC mean value differences were 1804 (95% CI 1191; 2417, p < 0.001) and 1198 (95% CI 593; 1802, p < 0.001) score points/hour for trials A and B, respectively. For the cough severity VAS, between-study heterogeneity was slightly higher than in case of the BSS (Fig. 2C).

### Cough intensity/frequency VRS

At baseline, more than 90% of the patients randomized to EA 575 or placebo in both studies described their cough as ‘frequent coughing that does not affect normal daily life or sleep’ (3 points) or as ‘serious coughing that is very frequent and interferes with normal daily life or sleep’ (4 points). These proportions decreased to 14.5% (33/227 patients) in the EA 575 group and to 37.0% (60/162 patients) at treatment day 7 (end of treatment). At the same visit, 100 patients (44.1%) in the EA 575 group reported mild, residual coughing during one short period without hardship (1 point) and 41 (18.1%) were symptom-free, compared to 38 (23.5%) and 15 patients (9.3%), respectively, in the placebo group. In the follow-up examination at two weeks after baseline (i.e., following one week without investigational treatment), 56.2% of the patients in the EA 575 group were cough-free and 32.3% had mild residual symptoms, compared to 25.6% and 33.1% of the patients treated with placebo. In the meta-analysis, scores were significantly lower in the EA 575 group as compared to placebo (Table 3), and the same applied to Studies A and B analyzed separately.

**Table 3:** Cough intensity/frequency VRS, global self-ratings of well-being and therapeutic effect—meta-analysis and individual trial results (full analysis set; p-values from ordinal logistic regression analysis).

Outcome	Visit	Patients with 1 or 0 score points^a^		Treatment group difference: p	Heterogeneity: p
		EA 575	Placebo		
**Meta-analysis**					
Cough frequency VRS	Treatment end	141 (62.1%) n = 227	53 (32.7%) n = 162	< 0.001	0.093
	Follow-up	200 (88.5%) n = 226	94 (58.8%) n = 160	< 0.001	0.209
Global assessment of well-being	Treatment end	184 (81.1%) n = 227	74 (45.7%) n = 162	< 0.001	0.056
	Follow-up	201 (88.9%) n = 226	82 (51.3%) n = 160	< 0.001	0.099
Global assessment of therapeutic effect	Treatment end	182 (80.2%) n = 227	65 (40.1%) n = 162	< 0.001	0.029
	Follow-up	188 (83.2%) n = 226	64 (40.0%) n = 160	< 0.001	0.138

Outcome	Visit	Patients with 0 or 1 score points^a^		Treatment group difference: p	
		EA 575	Placebo		
**Trial A**					
Cough frequency VRS	Treatment end (day 7)	56 (63.6%) n = 88	23 (25.0%) n = 92	< 0.001	
	Follow-up (day 14)	77 (87.5%) n = 88	51 (56.6%) n = 90	< 0.001	
Global assessment of well-being	Treatment end (day 7)	76 (86.4%) n = 88	36 (39.1%) n = 92	< 0.001	
	Follow-up (day 14)	79 (89.8%) n = 88	42 (46.7%) n = 90	< 0.001	
Global assessment of therapeutic effect	Treatment end (day 7)	75 (85.2%) n = 88	33 (35.9%) n = 92	< 0.001	
	Follow-up (day 14)	77 (87.5%) n = 88	31 (34.4%) n = 90	< 0.001	

Outcome	Visit	Patients with 0 or 1 score points^a^		Treatment group difference: p	
		EA 575	Placebo		
**Trial B**					
Cough frequency VRS	Treatment end (day 7)	85 (61.1%) n = 139	30 (42.9%) n = 70	< 0.001	
	Follow-up (day 14)	123 (89.2%) n = 138	43 (61.5%) n = 70	< 0.001	
Global assessment of well-being	Treatment end (day 7)	110 (79.1%) n = 139	35 (50.0%) n = 70	+ < 0.001	
	Follow-up (day 14)	123 (89.2%) n = 138	42 (60.0%) n = 70	< 0.001	
Global assessment of therapeutic effect	Treatment end (day 7)	107 (76.9%) n = 139	31 (44.2%) n = 70	< 0.001	
	Follow-up (day 14)	112 (81.2%) n = 138	34 (48.6%) n = 70	< 0.001	

*VRS* Verbal rating scale.

^a^Cough intensity/frequency VRS: 1 = ‘one short, mild period’ or 0 = ‘no cough’; global well-being and efficacy assessment: 1 = ‘well’ or 0 = ‘very well’.

### Global well-being and efficacy rating

At treatment end, the proportions of patients who reported feeling well or very well while undergoing treatment with the investigational product were 81.1% (184 of 227) for EA 575 and 45.7% (74 of 162) in the placebo group. In both groups these proportions increased slightly at end of follow-up (Table 3). In line with the global assessment of well-being, treatment efficacy of EA 575 was assessed as good or very good by 80.2% (end of treatment) and 83.2% (follow-up) of patients, compared to 40.1% (end of treatment) and 40.0% (follow-up) of patients of the placebo group (Table 3). In the meta-analysis EA 575 was significantly superior to placebo for all global ratings analyzed.

For the global ratings, notably for those obtained at treatment end (Day 7), meta-analysis results indicated heterogeneity between the two trials. It is worth mentioning, however, that the rate differences in both individual studies always favored EA 575.

### Safety

Adverse events were assessed by pooling the data from both trials rather than by performing meta-analyses. Across both studies, 30 of 228 patients (13.2%) randomized to EA 575 and 25 of 162 (15.4%) randomized to placebo experienced at least one adverse event (AE). The most frequently reported event was worsening of coughing which occurred in 21 patients (9.2%) treated with EA 575 and in 17 (10.5%) in the placebo group. Based on MedDRA Preferred Terms, the only other AE that was observed more than once in one group was sinusitis (EA 575 2, placebo 1). No serious or severe AEs were observed.

## Discussion and conclusions

Acute cough is a principal symptom of ARTIs such as acute bronchitis and the common cold. Even though such conditions are predominantly trivial and self-limiting, coughing may persist for several weeks and may cause considerable suffering and impairment of general well-being^11,12^. The aim of currently available, symptomatic treatment options is therefore to reduce the patients’ symptom burden and to accelerate the restoration of their health and daily living skills. In this context, the current disease management consensus guidelines of the German Respiratory Society for diagnosis and treatment of adults suffering from acute, subacute and chronic cough^32^ mention ivy leaves dry extract EA 575 as a phytopharmaceutical product whose protussive efficacy has been proven in randomized, controlled trials.

### Lessons learned

This IPD meta-analysis of two double-blind, randomized, placebo-controlled clinical trials demonstrated that EA 575, administered at daily doses of 105 mg for 7 days, lead to significant improvement of acute cough beyond the natural course of the underlying ARTI, which was observed in the BSS already after 2 days’ treatment. Across the study period, the decrease of the BSS total score in patients treated with EA 575 was always by about one visit ahead of that of patients who received placebo, e.g., on the fourth day of treatment patients in the EA 575 group exhibited an average BSS total score decrease comparable to that observed in the placebo group at day 7 (treatment end), and at treatment end the average score decrease in EA 575 treated patients was comparable to that observed for placebo at the end of the second week. As regards the magnitude of the observed difference to placebo, no empirically derived, minimal clinically important difference (MCID) has yet been published for the BSS, but according to Hedges’ g (g = 0.77) the criteria for a large treatment effect were missed only very narrowly in the meta-analysis. Moreover, in a responder analysis, the proportion of patients who achieved a subjectively detectable improvement of symptoms in the BSS was significantly higher in those who received EA 575 as compared to placebo.

The BSS results were supported by those for the cough severity VAS for which the magnitude of the treatment effect measured by the AUC across the entire treatment phase was comparable to the effect size for the AUC of the BSS.

Overall, these results thus indicate that treatment with EA 575 leads to a significant and important decrease of cough-associated symptoms and to an acceleration of recovery from acute cough over its natural course. These results are supported by those of a recent analysis of health survey data that showed that adults with ARTIs treated with EA 575 had a shorter duration of sick leave than those who received antibiotic medication instead^33^.

The verbalizations referring to the score points of the patient-rated cough VRS used in Studies A and B present a combination of coughing severity and frequency (e.g., ‘serious coughing that is very frequent and interferes with normal daily life or sleep’, corresponding to a score of four points out of a maximum of five). The VRS meta-analysis results indicate significant superiority of EA 575 over placebo and thus support the results for the BSS and the cough severity VAS. Interestingly, only about 40% of the patients in the EA 575 group, in contrast to about 70% in the placebo group, still showed perceivable coughing at treatment end, and 11% versus 41%, were still not cough-free two weeks after baseline. This is in line with findings from previous research according to which acute cough may persist for more than two weeks, even after other symptoms of the underlying ARTI have already subsided^18^. It also shows, however, that treatment with EA 575 may substantially reduce the proportion of patients who still cough after two weeks. Given the economic impact caused by ARTIs through reduced productivity or medical leave^13,14^, the shortening and alleviation of disease-related symptoms in not only beneficial for the patient but also advantageous economically, particularly when it also reduces the risk of complications and exacerbations to potentially more serious conditions^19^. Moreover, the results indicate that beneficial effects of EA 575 were not only observed in investigator-rated scales but also in patient-reported outcomes, and were thus also detectable for the patients subjectively.

It was interesting to note that for all outcomes investigated in our meta-analysis significant advantages of EA 575 over placebo were not only observed during and at the end of treatment, but also after untreated follow-up at the end of the second week. Of note, most of the treatment effect over placebo observed at end of treatment was still preserved at end of follow-up. The results thus indicate that the effect of one weeks’ treatment with EA 575 is not absorbed by the natural course of the underlying condition, but leads to persisting amelioration of coughing as well as to a significant acceleration of the recovery process.

AEs observed in patients treated with EA 575 were neither more frequent nor of a different type than those which occurred in the placebo group. Our analysis thus did not reveal any safety signals regarding EA 575.

We would like to emphasize that our investigation was not intended to be a systematic review into the efficacy of ivy leaves dry extracts in ARTIs, but an IPD meta-analysis of two pre-defined clinical trials performed in similar patient populations and according to very similar protocols^29,30^. An updated systematic review of clinical trials investigating mono and herbal combination products containing ivy leaf extracts has been presented by Sierocinski et al.^22^ in 2021. Among the 11 studies that met the authors’ eligibility criteria, only six of which were randomized, controlled trials, the two studies included into this meta-analysis were the only ones for which a low risk of bias was determined according to the Cochrane Collaboration’s bias assessment criteria^34,35^. A narrative review of the efficacy of EA 575 in the treatment of respiratory disorders published by Lang et al.^28^ included ten controlled clinical trials and eight observational studies. However, since all clinical trials reviewed by Lang et al. were performed in distinctly different indications (e.g., bronchial asthma, chronic bronchitis, COPD) and/or in different patient populations (e.g., children) and used different outcomes for assessing efficacy, their data was not considered feasible to be included into the current meta-analysis. Our work thus adds to what is already known by presenting the first quantitative synthesis of results from rigorously designed, randomized, placebo-controlled trials with EA 575, and by increasing the precision of the population estimates for the expectable effect sizes over that of each individual trial through the application of appropriate meta-analytical methods.

It is also important to note in this context that, due to differences in the origin of the medical plants, extraction methods, standardization, and manufacturing processes, different extracts from the same species, let alone combination preparations with other plants, are not interchangeable when reviewing the efficacy and safety of phytopharmaceutical products^32^. In addition to issues of methodological quality, this may also contribute to the heterogeneity of the study results observed by Sierocinski et al.^22^, which may in turn have impacted their over-all assessment of the effect of ivy leaf extract preparations. Reviews and meta-analyses investigating the effects of products based on the same medical plant may therefore be of limited value when differentiation between specific extracts is lacking.

### Limitations

The objective of this investigation was to provide summary evidence from the existing trials investigating the effect of EA 575 liquid on cough in compliance with current scientific standards. The meta-analysis results might have been more informative, had there been more than two eligible studies. It is also a certain limitation that these studies were performed by essentially the same research team.

Studies based on samples cannot be expected to yield identical results even when the underlying patient population from which they were taken is identical and the study protocols are comparable. Our IPD meta-analyses revealed only limited heterogeneity between Trials A and B. Where appreciable heterogeneity was observed, it was always caused by differences regarding the magnitude of the observed treatment effect but never by differences regarding the direction or statistical significance of the effect. This was expected given the similarity between the protocols and the populations of the two trials. The application of methods of meta-analysis therefor appears to be justified.

### Over-all conclusions

Overall, the results of this IPD meta-analysis of two randomized, double-blind, placebo-controlled trials demonstrated that ivy leaves dry extract EA 575 was efficacious in reducing the intensity of acute cough associated with ARTIs in adult out-patients. Moreover, treatment with EA 575 led to a significant acceleration of recovery. Tolerability of the product was comparable to that of placebo.

## Methods

### Included studies: objectives, design, ethical conduct

This is an IPD meta-analysis of pre-selected studies investigating the efficacy and safety of an EA 575 liquid formulation in ARTIs characterized by cough. The two studies included into this meta-analysis (Study A, EudraCT No.: 2014-003590-41^29^; Study B, EudraCT No.: 2016-002426-37^30^) were double-blind, randomized, placebo-controlled, parallel-group, multicenter trials that were performed to confirm the efficacy of EA 575 liquid in reducing cough severity in adult patients suffering from acute cough. The protocols of the trials included comprehensive, traceable descriptions of the procedures used for blinding and randomization.

The selected studies were the only double-blind, randomized, placebo-controlled trials with EA 575 liquid in adults with ARTIs that had been published at the time when the meta-analysis was performed. A literature search based in the search strategy employed by Sierocinski et al.^22^ and completed by the end of February 2022 did not identify any other randomized, placebo-controlled trials with EA 575 in adults with ARTIs.

After determining their eligibility, participating patients were randomized to the investigational treatments and were treated for 7 ± 1 consecutive days, with visits performed on the second, third, fourth, and seventh (last) day of treatment. A final follow-up visit was scheduled at two weeks after baseline.

The protocols of both trials were reviewed and approved by the competent authority (Bundesinstitut für Arzneimittel und Medizinprodukte/BfArM, Germany; reference numbers: study A: 61-3910-4040217, study B: 61-3910-4041585). A favourable opinion from the ethics committee of the Ärztekammer Nordrhein (medical council North Rhine, Germany) was given for both trials (reference numbers: study A: 2014403, study B: 2016277). All subjects provided written informed consent. The principles of Good Clinical Practice and the Declaration of Helsinki were adhered to.

### Assessments

The primary outcome measure for efficacy in Study A was a 100-mm visual analog scale (VAS) on which participants were asked to self-rate the current intensity of cough between the extremes of ‘no cough’ (0 mm) and ‘extreme cough’ (100 mm). The same scale was used in Study B as a secondary endpoint. The Bronchitis Severity Score (BSS)^36,37^, which was used in both studies, is a validated, observer-rated scoring system that includes the symptoms cough, sputum production, rales/rhonchi, chest pain during coughing, and dyspnea, each of which is assessed on a five-point verbal rating scale (VRS) ranging from 0 (‘absent’) to 4 (‘very severe’). A total score (up to 20 score points) was determined by adding up the individual item scores. In Study B, the total score of the BSS was used as the primary outcome measure. Further efficacy outcomes included a one-item, six-point cough intensity/frequency VRS ranging from 0 (‘no cough’) to 5 (‘distressing, continuous coughing that did not stop for 24 h’). Furthermore, a two-item, global efficacy assessment (GEA) was obtained from the patient and assessing self-rated, overall well-being as compared to the start of treatment as well as a global rating of the therapeutic effect using a five-point VRS ranging from 0 to 4 points verbalized as ‘very well’ to ‘very poor’. While the cough severity VAS, the BSS and the cough intensity/frequency VRS were assessed at all visits, the GEA items were completed at the end of treatment and at end of the follow-up period. Safety was assessed based on occurrence of adverse events (AEs).

### Participants

Male and female out-patients between 18 and 75 years of age and of any ethnic origin were eligible for inclusion if they suffered from acute cough of any genesis (Study A) or from acute bronchitis (Study B), with symptoms persisting for 48–72 h prior to start of treatment. Moreover, eligible participants were required to present with a cough severity VAS score of at least 50, a BSS of at least ten points, and a cough intensity/frequency VRS score of at least two points at baseline. Specific exclusion criteria for both studies included allergic bronchial asthma, bronchial hyperreactivity, chronic bronchitis, or other chronic or inherited lung disease, a history of drug hypersensitivity, asthma, urticaria, other severe allergic diathesis and acute hay fever, gastrointestinal complaints, fever > 38.3 °C (Study B only), or treatment with drugs affecting the respiratory or the immune system within 7 days before study inclusion.

### Interventions

Study A was a two-arm trial during which the participants received daily 3 × 5 mL of EA 575 liquid or a matching placebo according to the randomization. In Study B, which was a four-arm trial, patients were randomized to receive daily either 3 × 5 mL or 2 × 7.5 mL of EA 575 liquid, or a matching placebo. For all patients randomized to EA 575 the total daily dose was thus 15 mL, corresponding to 105 mg of extract.

The random code for both studies was generated by an independent statistician using validated random number generator software. The random block size was withheld from the investigators before unblinding. Patients were randomized at a ratio of 1:1 in Study A and at a ratio of 2:2:1:1 (EA 575 3 × 5 mL, EA 575 2 × 7.5 mL, placebo 3 × 5 mL, placebo 2 × 7.5 mL) in Study B.

### Statistics

The analysis was performed based on a prospectively defined statistical analysis plan. Meta-analysis outcomes included the absolute change of the BSS total score between baseline and subsequent visits, the area under the curve (AUC) calculated for the BSS total score and for the cough severity VAS for the entire 7-day treatment period by means of the trapezoid rule, as well as the proportions of patients indicating only minor impairment (i.e., a score of 0 or 1) on the cough severity VRS, in the global assessment of well-being, and in the global assessment of the therapeutic effect. Data were evaluated based on the analysis data sets of the included trials made available by the manufacturer of EA 575, using individual patient data (IPD) meta-analysis with a one-step approach^38^ rather than a meta-analysis of aggregated measures such as mean values, standard deviations, or proportions: for the BSS, change between baseline and subsequent visits was analyzed by means of mixed models for repeated measures (MMRM) which included treatment, study, and visit as fixed effects, patient as a random effect, and the baseline value as a covariate. Heterogeneity was assessed by the treatment-by-study interaction^39^. The AUC for the BSS as well as for the cough severity VAS was compared between the treatment groups using analysis of covariance (ANCOVA) with treatment and study as fixed effects, the baseline value as a covariate, and the treatment-by-study interaction for the assessment of heterogeneity. Treatment effects for individual visits as well as for Studies A and B were determined from the MMRM and ANCOVA models using contrasts. Tabulations include adjusted (marginal) mean values and their standard errors (SEM) as well as p-values for treatment comparisons.

Hedges’ g, a standardized effect size measure, was computed for assessing the between-group effect size for BSS total score change between baseline and treatment end. Moreover, a responder analysis was performed on the BSS total score change from baseline, based on the criterion of a minimal detectable difference (MDD), which was assumed to correspond to at least 15% of the range of the score as proposed by the German Institute for Quality and Efficiency in Health Care^40^. For the BSS total score, which has a range of 21 discrete values (0–20), the MDD was assumed to be 4 points.

The cough intensity/frequency VRS as well as the global patient self-ratings of well-being and therapeutic effect were compared between the treatment groups using ordinal logistic regression with terms for treatment, study, and the treatment-by-study interaction.

Efficacy analyses applied to the full analysis set (FAS) of the original studies, which included all randomized patients who received an investigational treatment at least once.

For Study B, the analysis pre-defined in the protocol showed no appreciable difference between the efficacy results obtained for EA 575 3 × 5 mL and 2 × 7.5 mL, and for placebo 3 × 5 mL and 2 × 7.5 mL, respectively (seed Schaefer et al.^30^ for details). For the meta-analysis, we therefore decided to pool the different dosing schemes within EA 575 and placebo, respectively.

To facilitate the interpretation of the results, two-sided p-values ≤ 0.05 were considered to be descriptively significant.

## Data Availability

The datasets used and analysed during the current study are available from the corresponding author on reasonable request.
